# Prophages in Enterococcal Isolates from Renal Transplant Recipients: Renal Failure Etiologies Promote Selection of Strains

**DOI:** 10.1155/2014/514689

**Published:** 2014-07-03

**Authors:** Agnieszka Daca, Tomasz Jarzembowski, Jacek M. Witkowski, Ewa Bryl, Bolesław Rutkowski, Alicja Dębska-Ślizień

**Affiliations:** ^1^Department of Pathology and Experimental Rheumatology, Medical University of Gdańsk, Dębinki 7 Street, 80-211 Gdańsk, Poland; ^2^Department of Microbiology, Medical University of Gdańsk, Do Studzienki 38 Street, 80-227 Gdańsk, Poland; ^3^Department of Pathophysiology, Medical University of Gdańsk, Dębinki 7 Street, 80-211 Gdańsk, Poland; ^4^Department of Nephrology, Transplantology and Internal Medicine, Medical University of Gdańsk, Dębinki 7 Street, 80-952 Gdańsk, Poland

## Abstract

Infections caused by commensal bacteria may be fatal for the patients under immunosuppressive therapy. This results also from difficulty in identification of high risk strains. Enterococcal infections are increasingly frequent but despite many studies on virulence traits, the difference between commensal and pathogenic strains remains unclear. Prophages are newly described as important elements in competition between strains during colonization, as well as pathogenicity of the strains. Here we evaluate a difference in presence of pp4, pp1, and pp7 prophages and ASA (aggregation substance) gene expression in enterococcal isolates from renal transplant recipients (RTx) with different etiology of the end-stage renal failure. Prophages sequence was screened by PCR in strains of *Enterococcus faecalis* isolated from urine and feces of 19 RTx hospitalized at Medical University of Gdansk and 18 healthy volunteers. FLOW-FISH method with use of linear locked nucleic acid (LNA) probe was used to assess the ASA gene expression. Additionally, ability of biofilm formation was screened by crystal violet staining method. Presence of prophages was more frequent in fecal isolates from immunocompromised patients than in isolates from healthy volunteers. Additionally, both composition of prophages and ASA gene expression were related to the etiology of renal disease.

## 1. Introduction

For many years* Enterococcus* species were believed to be harmless to humans and considered medically unimportant. Recently, enterococci have become one of the most common nosocomial pathogens, causing mortality rate up to 61% [1]. In the last decade enterococci have been reported as the second most common cause of wound and urinary tract infection and the third most common cause of bacteremia.* Enterococcus faecalis*, often regarded as a normal commensal of intestinal tract [2], is increasingly considered as a cause of nosocomial infections in patients undergoing immunosuppressive therapy due to, for example, organ transplantation [3, 4]. This is attributed, for example, both to the acquisition of multidrug resistance and to virulence factors [5]. Renal transplant recipients (RTx) often suffer from various urological malformations, which additionally increase the risk of even life-threatening infections caused by, for example, enterococcal strains.

Colonization of urinary tract by enterococci is epidemiologically associated with ASA (aggregation substance). The ASA encoded protein increases enterococcal adherence [6–8] and protects from killing by polymorphonuclear leukocytes [9, 10]. Although its urovirulent action has not been confirmed, ASA protein participates by adherence in first step of biofilm formation. Apart from the previously described virulence determinants, it has been recently discovered that enterococci may possess bacteriophages integrated as lysogenic prophages. Prophages, thanks to the active mechanism of integration to bacterial chromosome and excision, are able to add some new traits to their basic genome leading to obtaining new features and contributing to their evolution [11]. This is an important element of competition between strains during colonization as well as pathogenicity of the strains. Additionally, some of the prophages are found to influence adherence of the strain to human tissues.

Here we compare the adherence potential of enterococcal strains colonizing patients with different nephropathies by screening the prophages prevalence and ASA gene expression. Biofilm formation ability of the strains was also analyzed as an expected outcome of the adherence properties of the strains.

## 2. Results and Discussion

The majority of the known virulence traits found in enterococci are involved in adherence to extracellular structures and biofilm formation, important processes in initiating colonization and infection of the host. However, little is known about the difference in virulence gene expression between strains. Additionally, previous studies of the incidence of asa1 gene in enterococcal isolates are contradictory. In our study apart from ASA gene expression measurement, we decided also to consider prevalence of enterococcal prophages pp1 and pp4, newly described elements involved in adherence of the bacterial strain. It should be also noticed that the presence of virulence factors or their association with a strain from a particular isolation source did not seem to result from clonal spread of a few enterococcal genotypes. In another study (Dicuonzo et al., 2001) the authors analyzed the PFGE patterns of* E. faecalis* collection and observed extreme genetic heterogeneity in isolates.

**Figure 1:**
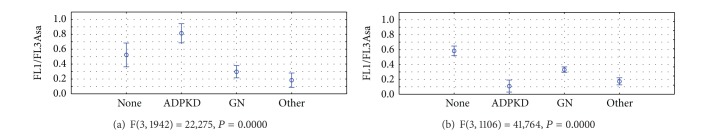
ANOVA comparison of ASA gene expression in enterococcal strains isolated from patients with different renal dysfunctions: ADPKD (autosomal dominant polycystic kidney disease), GN (glomerulonephritis), other (other nephropathies), and none (healthy volunteers). (a) planktonic cultures and (b) biofilm.

Here, the ASA gene expression, measured by FLOW-FISH, differs greatly in* Enterococcus faecalis *isolatesfrom patients with various renal dysfunctions and healthy people (Figure 1).

The significant difference was observed in ADPKD isolates. These strains presented the highest level of ASA gene expression in planktonic cells and the lowest level in biofilm. Although there is no evidence that ASA encoded protein may be involved in urinary tract infections so far [8], it influences colonization of urinary tract and is associated with the risk of endocarditis. ADPKD is the most common hereditary kidney disease. One of the most common complications of ADPKD is urinary tract infections (UTIs), with prevalence up to 60% [12]. ADPKD is mediated primarily by mutations in two different genes: PKD 1 and PKD 2 encoding polycystin 1 and polycystin 2, respectively [13]. Polycystins are, for example, engaged in basement membrane formation in renal tubules, so this abnormality may be associated with high ASA gene expression.

However, according to Creti et al. [14], the aggregation substance encoded by ASA gene is connected mostly with noninvasive infections. It is also present almost always in strains derived from healthy individuals. In our study we have also observed the higher level of ASA gene expression in biofilm formed by strains isolated from samples of healthy individuals than from samples of patients with UTI. It may be connected with the dependence observed, for example, by Creti et al. that the commensal strains, in contrast to the strains isolated from invasive and noninvasive infections, have always genes encoding aggregation substances. On the other hand, patients undergoing immunosuppressive therapy can have UTI without clinical symptoms (due to immunosuppression [4]) so recognition of UTI is always doubtful. Various authors are reporting that the enterococci participating in clinical infections express more of the virulence factors than enterococci in chronic, persistent cases [15] and healthy individuals [13]; this may suggest that the metabolic cost of expressing more genes (e.g., virulence genes responsible for antibiotic resistance) in the same moment may cause the lower level of expression of each virulence factor overall.

Besides the high ASA gene expression, enterococcal strains from patients with ADPKD differ from other end-stage renal diseases, taking into consideration the biofilm formation. The isolates from urine of ADPKD patients have the tendency for relatively low level biofilm formation (0,29–0,72; median OD—optical density—0,58), comparing with the ability for biofilm formation of other enterococcal strains isolated from urine of patients with other renal diseases (GN 0,33–1,07; median OD 0,72; other nephropathies 0,69–2,16; median OD 1,25) or commensal strains (0,26–2,27; median OD 0,86). Furthermore, the mass of biofilm produced by bacteria isolated from ADPKD patients' urine is lower than that produced by faecal isolates (0,39–1,52; median OD 0,88). The lack of such differences (in biofilm OD between the urine and feces enterococcal strains) in material from patients with other than ADPKD renal diseases suggests that the intestinal and urinary tracts' ecological niches of patients with ADPKD are more selective than in other compared groups of patients.

The presence of prophages in isolates was the third aspect analyzed in the study. Using the PCR technique, there was the prophages existence determined in* Enterococcus faecalis* strain V583. This strain belongs to the high-risk enterococcal clonal complexes, CC2, and it was hypothesized that V583 isolates are particularly well adapted to hospital environment and associated with invasive diseases due to richness in prophages elements [18]. Prophages are considered as the tool allowing bacteria to compete with other strains during colonization [16–18]. The horizontal transfer of additional genetic material gives the possibility to obtain new (and better) properties which allow bacteria to survive in a changing environment [18–20]. It is also suggesting a role of E.* faecalis* prophages in the development of nosocomial infective endocarditis. Prophages pp1 and pp4 are associated with the high adhesion. Third of studied prophages pp7 is defective and requires a helper phage to form infectious particles, but in contrast to pp4 it may produce infective virions. Additionally excision of pp4 is blocked at 37°C when pp1 is present. In all, temperate phages are likely to potentiate* E. faecalis* genetic and physiological flexibility for optimal adaptation during colonization or infection especially in biofilm.  Table 3 presents the phage sequence incidence in enterococcal isolates forming biofilm from urine/feces.

Higher incidence of prophages sequences in strains isolated from urine of patients during immunosuppression than of healthy people was observed (about 96%versus 30%). Such observation may be the evidence of high competition between strains in immunosuppression when colonization is poorly controlled by the host. This statement is also supported by more detailed analysis: unlike enterococcal isolates from healthy individuals, the prophages incidence in fecal isolates of immunocompromised patients was similar in strains isolated from urine.

Prophages' profile was also varied depending on the cause of renal failure. In patients who experience nephritis, incidence level of pp1 and pp4 sequence (linked to adhesion) was increased (Table 1), while in patients with ADPKD the most frequently occurring sequences were pp7 (Table 1).

**Figure 2:**
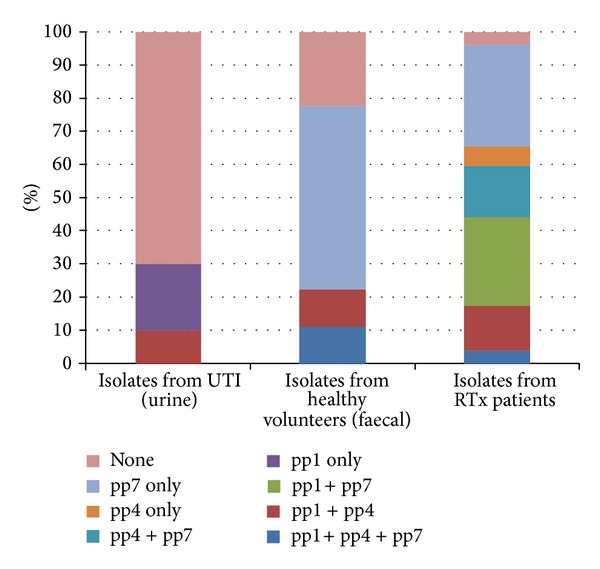
Composition of the prophages in isolates. The significance of difference between all groups was confirmed by ANOVA analysis on *P* = 0.0006.

The frequency of prophages occurrence in enterococcal isolates is also differential if the tendency to develop symptomatic or asymptomatic (due to undergoing immunosuppression) bacteriuria is taken into consideration. As shown in Figure 2, the prophages profile is completely different depending on the lack or existence of symptoms of infection. The prophages profile is also different in bacteria isolated from healthy volunteers (Figure 2).

The contrast in prevalence of pp1-, pp4-, and pp7-strains from RTx patients and isolates from urine in UTI may be explained by high competition between strains in immunocompromised patients. Such statement is also supported by higher prevalence of prophages in faecal isolates. Isolates from RTx patients were also unique by presence of pp4 prophages alone and pp7 prophages. However, in all groups adherence-related strains were present.

Different etiology of end-stage renal failure is related with difference in pretransplantation treatment and risk of infection. As shown in Figure 1, the relation between the ASA gene expression and the type of end-stage renal failure varies considering the type of disease. The level of ASA gene expression in planktonic cultures is almost the same in bacteria isolated from patients with all diseases leading to end-stage renal failure except ADPKD, where that level is about 3-fold higher. ADPKD is a disease with complex etiology. In its course, high levels of apoptosis and proliferation [21, 22] and frequent infections, for example, are observed [23, 24]. It is also the only disease (amongst our patients) with genetic undercurrent [21]. The difference in ASA gene expression may be due to different treatment protocol of patients with ADPKD. They were treated with tacrolimus, in contrast to patients with other end-stage renal failures. That could result in an increase of gene expression as was described by authors previously in the context of PBP5 (penicillin binding protein 5) gene expression [25]. It may lead to selection of commensal* Enterococcus* strains with higher ASA gene expression.

The other features, which distinguish bacteria isolated from patients with various renal-related diseases, are the existence of different prophages in isolates from their urinary and intestinal tracts. As presented in Table 2., patients with immunosuppression are characterized by colonization with bacteria exhibiting the presence of different types of prophages than patients without immunosuppression but currently with bacterial infection and healthy volunteers (Figure 2).

To conclude, all results presented above support statement that both immunosuppressive therapy and etiology of renal-related diseases have selective potential, allowing only bacteria with particular features (ASA gene expression, biofilm formation ability, and specific prophages coexistence) to colonize intestinal and/or urinary tract. What is more, in our opinion, strains with the low ASA expression in biofilm and/or pp1(+) pp7(+) phenotype should be considered as high-risk strains. However, further in vivo analysis is necessary to confirm this conclusion.

## 3. Material and Methods

Forty-four enterococcal strains were isolated from urine and feces of nineteen RTx patients hospitalized at the Medical University of Gdańsk. All patients initially underwent induction with monoclonal (basiliximab) or polyclonal antibodies (ATG) and were prescribed subsequently tac (tacrolimus) + MMF (mycophenolate mofetil)/MPS (mycophenolate sodium) + glucocorticosteroids or CsA (cyclosporine) + MMF/MPS + glucocorticosteroids or CsA + everolimus + glucocorticosteroids. More detailed characterization of patients is presented in Table 2.

As a reference group, 18 enterococcal strains of* Enterococcus faecalis* were isolated from healthy voluntaries. The isolates were identified to species level by strep ID test (BioMerieux) and classified as different strains of* Enterococcus faecalis *by biochemical and resistance profiles. The strains were cultured in BHI medium at 37°C for 18 h.

To evaluate ASA gene expression by the FLOW-FISH method, we used a linear locked nucleic acid (LNA) probe, AGCGATAAACTAGACGTCAAAC-ATGACA 5′FITC, containing nucleic acid analogs with higher affinity for DNA and RNA [26]. As a positive control, Enfl84 probe (3′-ACGTGAGTTAACCTTTCTCC) [27] targeting 16srRNA gene was used. Oligonucleotides were synthesized commercially (Metabion, Germany) and labeled with fluorescein isothiocyanate (FITC). For hybridization, the procedure described by Waar et al. [27] was adopted and modified [28]. Briefly, cell membranes were permeabilized by incubation for 30 min at 37°C in permeabilisation buffer (Tris-EDTA) consisting of 1 mg/mL lysozyme (DNA Gdansk, Poland). Then, the cells were suspended in 1 mL of 0.9% NaCl and sonicated for 2 minutes on ice. To ensure permeabilisation of the cells, the propidium iodide (PI, 1 *μ*g/mL) staining of DNA was used. Particles without PI fluorescence (FL3) were excluded from further investigation. Fluorescence of particles was determined using a FACScan flow cytometer (Becton-Dickinson, Franklin Lakes, NJ, USA). The mean probe fluorescence (FL1) normalized by DNA fluorescence (FL3) and the median fluorescence (MFL1) weighted by percentage of probe-binding particles (FL1 positives) were analyzed. Results were tested by analysis of variance (ANOVA) by StatSoft software (Statistica 10, USA).

Bacterial DNA was isolated using a commercially available kit (A&A Biotechnology, Poland). The presence of pp1, pp4, and pp7 prophages sequences was detected by the PCR method, as described earlier [9].

PCR was performed in a 50 mL reaction mixture that consisted of template DNA, 20 pmol of each primer (Table 3), and Hypernova-RED master mix (DNA Gdańsk) in a Biometra thermocycler (Biometra, Germany). Sample without DNA was used as a negative control. Denaturation lasted for 2 min at 94°C, annealing at Tm for 30 s, and elongation at 72°C for 2 min. Results were visualized on 2% agarose (Prona Marine Research Institute, Spain) stained with ethidium bromide.

Biofilms of these strains were formed in flat-bottom wells (TRP, Switzerland). The amount of the biofilm was estimated by crystal violet staining (0.1%).

## Figures and Tables

**Table 1:** The frequency of occurring prophages sequences in bacteria from different materials (feces versus urine) isolated from various groups of people (with ADPKD, glomerulonephritis (GN), other nephropathies, and healthy volunteers).

Prophages	pp4	pp1	pp7	pp(−)	pp(+)
Material from healthy volunteers					
Urine	*30,00% *	*10,00% *	*0,00% *	70,00%	*30,00% *
Feces	22,22%	22,22%	66,67%	22,22%	77,78%
Material from patients with immunosuppression					
Urine	**42,86%**	**35,71%**	**78,57%**	3,57%	**96,43%**
Feces	**45,83%**	**45,83%**	**70,83%**	4,17%	**95,83%**
Causes of end-stage renal disease					
ADPKD	38,46%	7,69%	92,31%	7,69%	92,31%
GN	52,38%	52,38%	76,19%	0,00%	100,00%
Others	38,89%	50,00%	61,11%	5,56%	94,44%

**Table 2:** Characteristic of patients.

Number of patients	Years after transplantation	Comorbidity	Number of UTI	Cause of renal failure
19	1.08 ± 1.03	4.42 ± 1.74	0–7 (0: 12p.∗, 1: 3p., 2: 0p., 3: 0p., 4: 1p., 5: 1p., 6: 1p., 7: 1p.)	ADPKD∗∗: 4 patients; glomerulonephritis: 8 patients; others∗∗∗: 7 patients

∗p: patients.

∗∗ADPKD: autosomal dominant polycystic kidney disease.

∗∗∗others: diabetic and hypertensive nephropathy and tubulointerstitial and lupus nephritis, mean ± SD.

**Table 3:** Primers used for PCR.

pp1F	GCAGTACAGATTATAAAA
pp1R	GATCGGCAACAAGTAATGTC
pp7F	ACAGCACCAGACCCGACAG
pp7R	ACGACGAGGTTCCATGTGATG
pp4F	CAGTTCGAGTCCTGTATGG
pp4R	AGAACGGCTTTTCAGAGAAG
